# Cannabidiol improves Nile tilapia cichlid fish welfare

**DOI:** 10.1038/s41598-022-21759-3

**Published:** 2022-10-21

**Authors:** Bruno Camargo-dos-Santos, Marina Sanson Bellot, Isabela Inforzato Guermandi, João Favero-Neto, Maira da Silva Rodrigues, Daniel Fernandes da Costa, Rafael Henrique Nóbrega, Renato Filev, Eliane Gonçalves-de-Freitas, Percília Cardoso Giaquinto

**Affiliations:** 1 Department of Structural and Functional Biology, Institute of Biosciences of Botucatu, São Paulo State University (UNESP), Botucatu, SP 18618-689 Brazil; 2 CAUNESP – Aquaculture Center of UNESP, São Paulo State University, Jaboticabal, SP 14884-900 Brazil; 3 Reproductive and Molecular Biology Group, Department of Structural and Functional Biology, Institute of Bioscience, São Paulo State University (UNESP), Botucatu, SP 18618-970 Brazil; 4 Department of Psychiatry and Medical Psychology, Escola Paulista de Medicina, Universidade Federal de São Paulo, São Paulo, SP 04017-030 Brazil; 5 Departamento de Ciências Biológicas, Instituto de Biociências, Letras e Ciências Exatas, São Paulo State University (UNESP), São José do Rio Preto, SP 15054-000 Brazil

**Keywords:** Animal behaviour, Animal physiology, Reproductive biology

## Abstract

Cannabidiol (CBD) is a substance derived from *Cannabis sativa*, widely studied in medicine for controlling neural diseases in humans. Besides the positive effects on humans, it also presents anxiolytic proprieties and decreases aggressiveness and stress in mammals. Therefore, CBD has the potential to increase welfare in reared animals, as it seems to reduce negative states commonly experienced in artificial environments. Here, we tested the effect of different CBD doses (0, 1, 10 and 20 mg/kg) on aggressiveness, stress and reproductive development of the Nile tilapia (*Oreochromis niloticus*) a fish reared worldwide for farming and research purposes. CBD mixed with fish food was offered to isolated fish for 5 weeks. The 10 mg/kg dose decreased fish’s aggressiveness over time, whereas 20 mg/kg attenuated non-social stress. Both doses decreased the baseline cortisol level of fish and increased the gonadosomatic index. However, CBD 1 and 10 mg/kg doses decreased the spermatozoa number. No CBD dose affected feeding ingestion and growth variables, showing that it is not harmful to meat production amount. Despite the effect on spermatozoa, CBD supplementation exhibits high potential to benefit animals’ lives in artificial environments. Therefore, we showed for the first time that CBD could be used as a tool to increase non-mammal welfare, presenting a great potential to be explored in other husbandry and captivity species.

## Introduction

The science of animal welfare has been growing this century, as several studies showed the importance of meeting animal needs to ensure a better quality of life^1–3^. Most researchers consider that animals are at a good welfare level when they are able to adapt to their current artificial environment without suffering from the conditions provided by humans^4–6^. The suffering is related to animals’ “affective states”, and could be challenging to investigate directly^4^. However, many indirect indicators can be used to evaluate if animals are suffering or not in their current environments, such as physiological, behavioral and histological measures^4^. Thus, investigating ways to mitigate the effects of artificial environments by reducing stress and unpleasant emotional states in animals is a serious and urgent concern, especially in species widely farmed and at serious risk of compromised welfare.

The Nile tilapia (*Oreochromis niloticus*) (Linnaeus, 1857) is an African cichlid reared worldwide for farming and research purposes^7^. Nile tilapia production has been growing exponentially in the last few years, and the species has become the third most farmed fish in the world, with global production reaching approximately 4407.2 tons in 2020^8^. The intensification of Nile tilapia aquaculture required stressful handlings for fish^9^ and relies on maintaining fish in high stock densities^10^. Therefore, even being one of the most important fish species for the aquaculture industry, Nile tilapias face several suboptimal conditions in their farming process that can affect their welfare.

The Nile tilapia is an aggressive and territorial species, presenting a social-rank-based dominance hierarchy^11^. In aquaculture, Nile tilapias aggressively interact with each other for territories inside the tanks, establishing social hierarchies. These social hierarchies organize the access to food, and dominant fish eat before the subordinates, reducing some costs of prolonged fights^11^. These social ranks are kept among fish through visual^12,13^, chemical^14^, and acoustic cues^15^. However, aquaculture environments usually compromise these signs, especially the high density, leading to an impairment of social ranks and increased aggressive interactions between individuals^11^. The higher the aggressive interactions, the higher the social stress from social rank, the energy expenditure of fights, and the probability of body injuries, infections, and deaths^11^. In addition, fish are sentient animals^16^, and, thus, is expected to be an increase in pain associated with increased injuries.

Besides compromising the social behavior of the Nile tilapia, aquaculture environments severely stress fish. Overall, stressors like changes in water quality^17^, handlings (e.g., grading, capturing, and transporting^9^), and high stock densities^18,19^ will trigger physiological responses, such as activation of the autonomic nervous system following rapid cardiac and respiratory adjustment (ventilation rate [VR] increase, for example), and epinephrine releasing, as well as activation of the Hypothalamus-Pituitary-Interrenal axis, culminating in increased cortisol levels, among other stress responses^19,20^. A chronic stress state can lead animals to non-adaptive behavioral and morphological alterations, such as decreasing reproductive performance^9,21^, reducing food intake, weight loss and impairment of the immune system^19,20^. Altogether, these behavioral and physiological alterations triggered by the aquaculture environments severally compromise Nile tilapia welfare. Therefore, finding strategies that improve fish welfare and mitigate as many as possible adverse effects of the rearing environment on Nile tilapia is of great importance.

A way to counteract the negative effects of the rearing environment in fish is by using chemical substances known to reduce stress and stimulate indicators of good welfare. For example, the amino acid tryptophan mixed in the food reduces aggressive behavior and stress in Nile tilapia^7^ and rainbow trout (*Oncorhynchus mykiss*^22,23^. Vitamin E, when added to food, reduces the stress on rainbow trout^24^ and pacu^25^ (*Piaractus mesopotamicus*) submitted to high densities. In addition, vitamin E also improves inflammatory response in pacu^25^. In turn, vitamin C in food decreases stress and prevents skeletal malformations in carps^26^ (*Cyprinus carpio*). Recently, a promising substance that has a great potential to increase the welfare of farm animals is one of the major cannabinoids from the *Cannabis sativa* plant, cannabidiol (CBD)^27^.

The CBD presents many pharmacological properties and great medicinal potential, assisting the treatment of many human diseases and psychiatric disorders^28^. CBD also present a wide range of effects in non-human mammals. In Wistar rats, CBD (10 mg/kg) shows anxiolytic-like effects in the Vogel conflict test^29^ and the social interaction test (1 mg/kg)^30^. In Swiss mice, CBD (30 mg/kg) induces antidepressant-like effects in the forced swimming test^31^, attenuates stress induced by restraint (1–20 mg/kg)^32^, increases the body weight gain in chronically stressed rats (10 mg/kg)^33^ and decreases aggressive behavior in the resident-intruder test (5–60 mg/kg)^34^. In addition, CBD also decreases the aggressive behavior of shelter dogs toward humans (CBD-based oil 5%)^35^ and presents anti-inflammatory effects in several rodents’ models^36^. Despite the many positive effects of CBD in the organism of non-human mammals, the drug presents some negative effects on the male reproductive system in rats, mice, and monkeys (e.g., reduction in testis size, and fertilization rates, among other effects. See^37^ for more details).

The mechanisms responsible for most CBD effects are still not completely clear. CBD acts in multiple targets and receptors, such as the serotoninergic system, by activating 5-HT1A receptors^34,38^ and the endocannabinoid system. Essentially, this is a neuromodulator system, which will allow or cease the neurotransmissions throughout the organism^39^. CBD acts indirectly on this system, blocking the fatty acid amide hydrolase enzyme (FAAH)^39,40^, responsible for degrading anandamide, one of the primary vertebrates endocannabinoids ligands^41^. Thus, CBD increases the anandamide supply in organisms and consequently actives CB1 and CB2 endocannabinoid receptors^34^.

The CBD decreases aggressiveness in mammals through a mechanism associated with activating both receptors, 5-HT1A and CB1^34^. The activation of 5-HT1A receptors by the drug is also related to the decrease in stress and anxiety^42,43^. The endocannabinoid receptors also have a regulatory effect on the Hypothalamic-Pituitary-Adrenal (HPA) axis, responsible for mediating stress responses in mammals^44^, besides having a key role in the food intake and body weight gain^45^. Moreover, the endocannabinoid system is involved in the regulation of male^46,47^ and female^48,49^ fertility.

The effects of CBD demonstrated in mammals are expected to be similar in other vertebrate groups, such as fish, since the 5-HT1A serotoninergic receptors and the endocannabinoid system are highly conserved between the taxa^50^. For example, fish CB1 receptors have about 70% similarity with CB1 receptors of rodents and humans^51^. Thus, it is expected that the CBD effects in mammals involving the activation of these two receptors are similar in fish. Indeed, some studies have already shown that in zebrafish (*Danio rerio*), CBD presents an anxiolytic effect^52^; decreases the natatory rhythm, stimulates differentiation and regulation of immunity genes^53^; and improves some reproductive parameters in females but shows reproductive toxicity for males^47^. These studies show the potential of CBD to improve fish welfare in captive environments, mainly in aggressive species.

**Figure 1:**
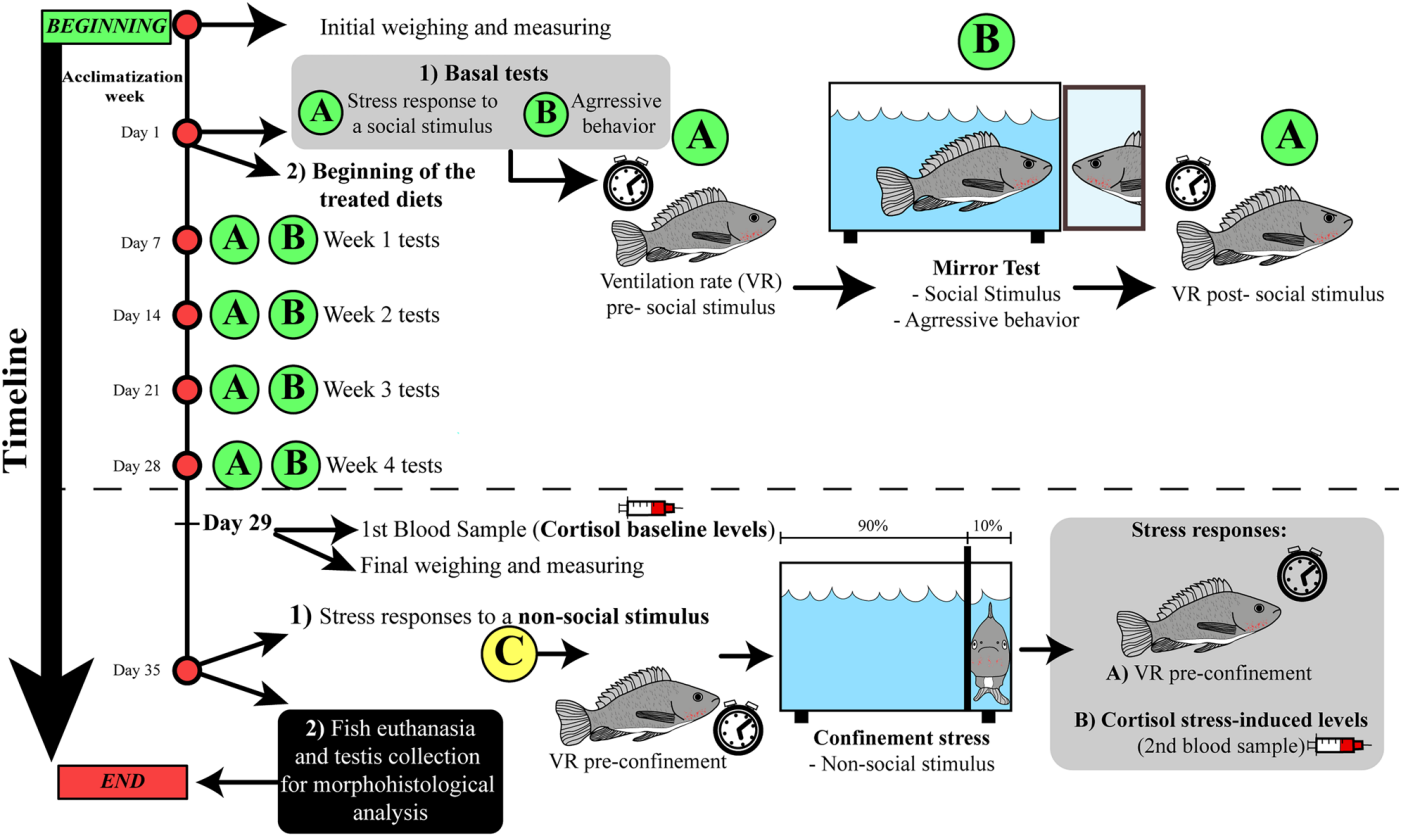
Experimental timeline and design—sequence of events during all the experiment (n = 15/group). The procedures start with fish acclimatizing to the isolated aquarium for 7 days (Acclimatization Week). Followed this step, firstly, on Day 1, basal fish’s aggressiveness level (represented in the figure by the green “A” letter) and stress response (Ventilation rate [VR]) to a social stimulus (represented in the figure by the green “B” letter”) were accessed through the mirror test. Afterward, fish started to receive the treated diets containing different cannabidiol (CBD) doses—0 (control), 1 (CBD 1), 10 (CBD 10) and 20 mg/kg (CBD 20). During the first four experimental weeks (Day 1 to Day 28), the aggressive behavior and the stress responses to a social stimulus were accessed once a week. In the fifth experimental week, some stress responses to a non-social stimulus (confinement stress) were measured (represented in the figure by the yellow “C” letter): on Day 29, blood samples were collected to analyze fish’s cortisol baseline levels; and on Day 35, confinement stress was applied to fish, and the VR and cortisol stress-induced levels were collected. Lastly, fish were euthanized (1500 µl/l of clove oil), and their testis were collected for morpho-histological analysis (gonads’ size and the number of spermatozoa).

Here, we tested the effect of CBD on aggressiveness, stress, and reproductive development in Nile tilapia. CBD’s effects on the organism vary according to the dose and can be both dose-dependent and biphasic^47,54^. For example, Ewing et al.^55^ tested the CBD effect in some gene expressions in mice’s livers. Most genes were upregulated in a dose-dependent manner (more expressed with higher doses of CBD), while others were upregulated at lower doses and downregulated at higher doses (biphasic effect)^55^. Therefore, different doses must be tested to assess whether the CBD effect on a determined variable will be dose-dependent or biphasic. We tested doses of 0 (control), 1 (CBD 1), 10 (CBD 10), and 20 mg/kg (CBD 20), which were mixed with food and offered to fishes for 5 weeks (Fig. 1). We hypothesized that the drug would affect fish’s aggressive behavior, stress responses, and male’s reproductive system because CBD can activate 5-HT1A serotoninergic receptors and/or CB1 and CB2 endocannabinoids receptors. Specifically, we predicted that high doses of CBD would increase fish welfare by decreasing animals’ aggressiveness and stress responses (specifically VR and cortisol levels) (similar doses presented efficient results in decreasing stress and aggressive behavior in other animal species^32,34^) and improving reproductive aspects, such as testis size and spermatozoa count.

## Results

### A high dose of CBD reduced aggressive behavior

The same test (mirror test—see “Methods” for more details) was used during the first four experimental weeks to assess fish’s aggressive behavior and stress response to a social stimulus (fish’s reflected image in the mirror) (Fig. 1). These responses were collected through five sampling time points: basal (the first day of experiment, before fish start to receive the treated diets) and once per week after the beginning of CBD treatment (Week 1, Week 2, Week 3 and Week 4; Fig. 1). To assess fish’s aggressive behavior, the frequency of direct attacks and the latency for the first aggressive behavior against the mirror were accounted for (see “Methods” for more details about the “Mirror Test”).

**Figure 2:**
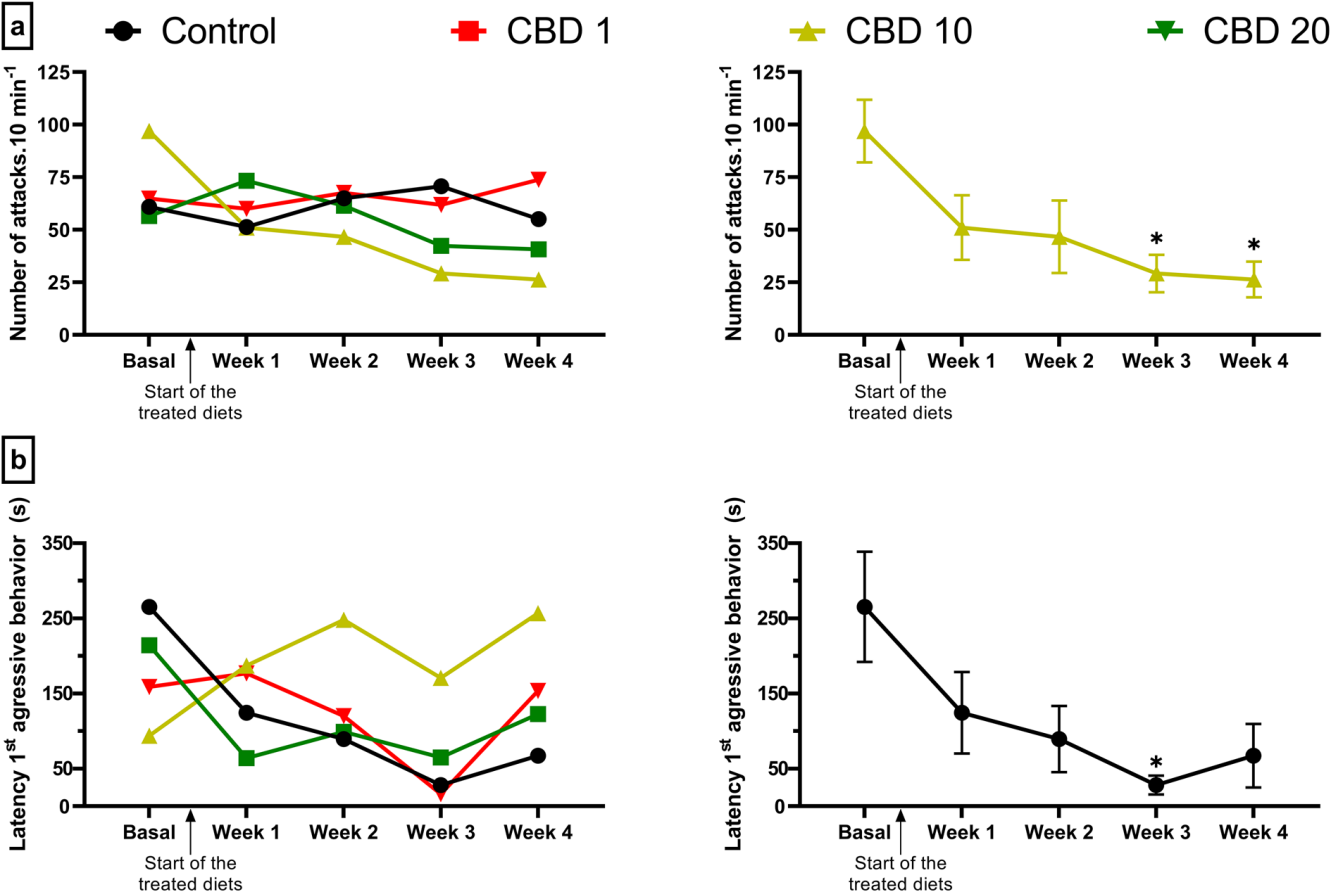
Effect of diets containing different cannabidiol (CBD) doses, 0 (control), 1 (CBD 1), 10 (CBD 10) and 20 mg/kg (CBD 20) on (**a**) the number of attacks and (**b**) the latency for the first aggressive behavior of Nile tilapias (n = 15). These response variables were evaluated over five sampling time points, Basal, Week 1, 2, 3 and 4. The arrow indicates the beginning of the application of the treated diets. The graphs on the left present the mean values without SEM of all CBD treatments over the five sampling time points, while the graphs on the right present the mean ± SEM values of only the treatments that presented significant differences over the sampling time points. The asterisks above a mean value indicate significant differences between this measurement and the baseline measurement of the same groups (Tukey HSD test, p < 0.05).

Fish from the CBD 10 treatment significantly decreased attacks over the sampling time points (Fig. 2a, Table 1). During the third and fourth weeks of CBD administration, fish from CBD 10 treatment attack the mirror less than in their basal measurement. This decrease in the number of attacks was not observed in the other treatments (Linear mixed model [LMM], treatment: F_3, 55.94_ = 0.362, p = 0.78; sampling time points: F_4, 222.1_ = 2.516, p = 0.042; treatment * sampling time points: F_12, 222.11_ = 2.424, p = 0.006; Tukey HSD test: CBD10 basal vs. Week 3: p < 0.001; CBD 10 basal vs. Week 4: p < 0.001; Fig. 2a).

**Table 1:** Short table summarizing the results found in this study.

Response variable	Control	CBD 1	CBD 10	CBD 20
Aggressive behavior	NS	NS	**Decreased#**	NS
Latency for the first aggressive behavior	**Decreased#**	NS	NS	NS
VR response to a social stimulus	NS	NS	NS	NS
VR response to a non-social stimulus	NS	NS	NS	Decreased*
Baseline cortisol levels	NS	NS	Decreased*	Decreased*
Non-social stress-induced cortisol levels	NS	NS	NS	NS
Feeding ingestion and growth variables	NS	NS	NS	NS
Gonadosomatic Index (GI)	NS	NS	Increased*	Increased*
Number of spermatozoa	NS	Decreased*	Decreased*	NS

*CBD* cannabidiol, *VR* ventilation rate.

‘NS’ indicates no significant effect of the group in the indicated response variable (Tukey HSD test, p > 0.05).

‘#’ indicates significant differences between the group and the baseline measurement of the same treatment (Tukey HSD test, p < 0.05).

‘*’ indicates significant differences between the group and the control (Tukey HSD test, p < 0.05).

Regarding the latency for the first aggressive behavior, fish from the control treatment decreased the latency for their first attack against the mirror over the time (Fig. 2b, Table 1). In the third week, fish from the control group performed their first attack significantly faster than their basal measurement. This pattern was not observed in any other treatment exposed to CBD (LMM, treatment: F_3, 56.266_ = 1.253, p = 0.299; sampling time points: F_4, 222.638_ = 3.03, p = 0.018; treatment * sampling time points: F_12, 222.644_ = 1.986, p = 0.027; Tukey HSD test: control basal vs. week 3: p = 0.042; Fig. 2b).

### CBD did not attenuate stress in response to a social stimulus

We tested whether different CBD doses could attenuate fish’s stress responses induced by their reflected image on a mirror (social stimulus)^56^. For this purpose, we measured animals’ ventilation rates (VR) before (pre-social stimulus) and after (post-social stimulus) the mirror test (Fig. 1). Afterward, we measured the individual variation of VR in response to the mirror test (ΔVR = VR post-social stimulus–VR pre-social stimulus) as our main dependent variable (see “Methods” for more details about the VR measurement).

We did not observe a significant effect on treatments, and sampling time points; neither interaction between treatments and sampling time points on the ΔVR (LMM, treatment: F_3, 60_ = 1.929, p = 0.134; sampling time points: F_4, 240_ = 0.867, p = 0.484; treatment * sampling time points: F_12, 240_ = 0.62, p = 0.824; Table 1).

### A High CBD dose reduced fish’s VR response to a non-social stimulus

In the fifth week of CBD administration, we tested if the drug could attenuate fish’s stress responses (namely VR and cortisol levels) to a confinement stressor (non-social stress) (Fig. 1). We measured animals’ VR pre-confinement and VR post-confinement to calculate fish’ VR variation (ΔVR) in response to confinement stress. In addition, we measured fish’ baseline cortisol levels (without applying stress) and the stress-induced cortisol levels (after applying confinement stress in fish) (see “Methods” for more details about VR and cortisol).

**Figure 3:**
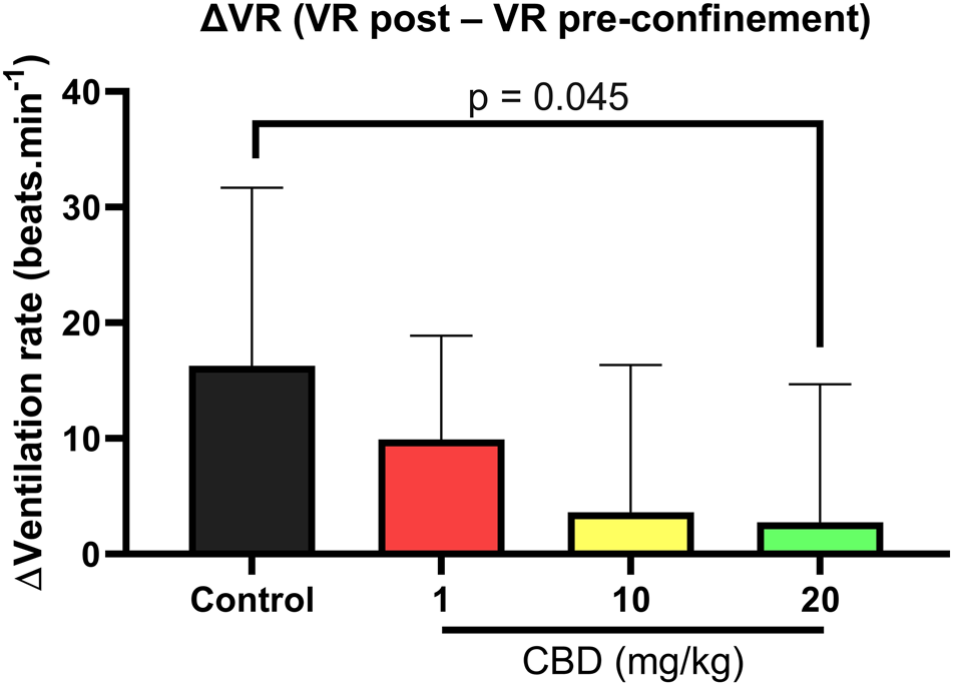
Effect of diets containing different cannabidiol (CBD) doses, 0 (control), 1 (CBD 1), 10 (CBD 10) and 20 mg/kg (CBD 20) on the ΔVR (VR post–VR pre-confinement) of Nile tilapias (n = 14). The stress applied was a confinement stressor (non-social stress). The highlighted p-value indicates a significant difference between treatments under the Tukey HSD test (p < 0.05). Values are mean ± SEM.

We observed a significant effect of CBD on the ΔVR (Table 1). Fish from CBD 20 treatment varied less the VR after confinement stress, presenting a lower ΔVR compared to control fish (one-way ANOVA, F_3, 45_ = 3.08, p = 0.037; Tukey HSD test: CBD 20 vs. Control: p = 0.045; Fig. 3).

### High CBD doses decreased the baseline but not the stress-induced cortisol levels

**Figure 4:**
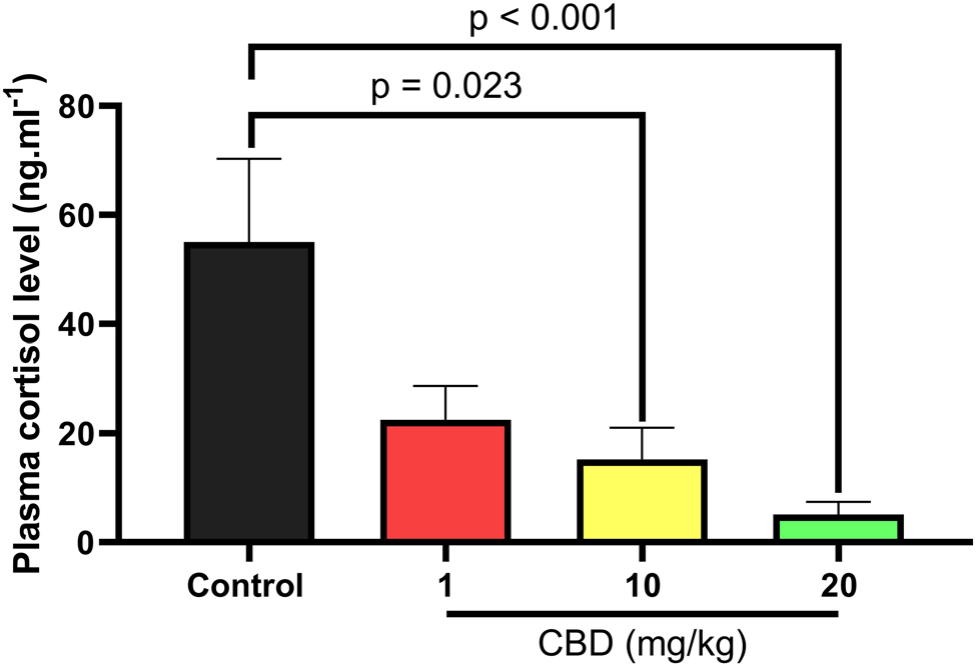
Effect of diets containing different cannabidiol (CBD) doses, 0 (control), 1 (CBD 1), 10 (CBD 10) and 20 mg/kg (CBD 20) on the baseline plasma cortisol levels of Nile tilapias (n = 8). Fish received the treated diets for 28 days before the baseline measure was collected. Highlighted p-values indicate a significant difference between treatments under the Tukey HSD test (p < 0.05). Values are mean ± SEM.

The baseline cortisol levels of fish from CBD 10 and CBD 20 groups were lower than control group (one-way ANOVA, F_3, 24_ = 7.621, p < 0.001; Tukey HSD test: CBD 10 vs. Control: p = 0.023; CBD 20 vs. Control: p < 0.01; Fig. 4; Table 1). However, in relation to the stress-induced cortisol levels, we did not observe a significant effect of CBD (one-way ANOVA, F_3, 27_ = 1.127, p = 0.356, Table 1).

### CBD did not affect feeding ingestion and growth variables

During the acclimatization week and the first four experimental weeks, fish feed ingestion and the following growth variables were measured: average weight gain (AWG), feed conversion (FC), specific growth rate (SGR), and condition factor (K). In addition, feeding ingestion was also measured in the fifth week and analyzed apart from other weeks due to fish manipulation for blood sample collection (see “Methods” for more details).

The feed ingestion during the acclimatization week did not differ between the treatments (H_3_ = 5.236, p = 0.155). Moreover, CBD did not decrease fish feed ingestion during the first four experimental weeks. Independent of the CBD dose received, fish decreased their feed ingestion in the last experimental weeks (LMM, treatment F_3, 60_ = 0.641, p = 0.591; sampling time points: F_3, 180_ = 5.304, p = 0.002; treatment * sampling time points: F_9, 180_ = 0.892, p = 0.533; Tukey HSD test: week 1 vs. week 3: p = 0.034; Week 1 vs. Week 4: p = 0.003; Week 2 vs. Week 4: p = 0.029). Fish feeding ingestion was also unaffected by CBD in the fifth experimental week (Kruskal–Wallis test, H_3_ = 3.084, p = 0.379).

In addition, no growth variable analyzed was affected by CBD (AWG: Kruskal–Wallis Test, H_3_ = 0.37, p = 0.946; FC: Kruskal–Wallis Test, H_3_ = 0.128, p = 0.988; SGR: one-way ANOVA, F_3, 55_ = 0.096, p = 0.962 and condition factor [K]; Kruskal–Wallis Test, H_3_ = 1.757, p = 0.624; Table 2).

**Table 2:** Growth response variables of Niles tilapias fed during 28 days with diets containing different cannabidiol (CBD) doses.

Treatments	AWG (g)	FC (g/g)	SGR (%)	K (%)
Control	17 ± 7.512	1.742 ± 0.895	1.242 ± 0.492	3.483 ± 0.484
CBD 1	18 ± 7.746	1.737 ± 1.158	1.337 ± 0.534	3.476 ± 0.481
CBD 10	16.357 ± 6.935	1.707 ± 0.395	1.37 ± 0.369	3.503 ± 0.477
CBD 20	19.545 ± 8.409	1.681 ± 0.983	1.286 ± 0.489	3.454 ± 0.507

The absence of asterisks indicates there is no significant difference between the treatments under one-way ANOVA or Kruskal–Wallis tests.

Values are mean ± SD (n = 15).

No significant differences between the treatments under one-way ANOVA or Kruskal–Wallis tests.

*AWG* average weight gain, *FC* feed conversion, *SGR* specific growth rate, *K* condition factor, *CBD* cannabidiol.

### CBD increased testis size but decreased the number of spermatozoa

At the end of the fifth experimental week, fish were euthanized, and the testis were collected for morpho-histological analyzes (Fig. 1). Right after being collected, the gonads were weighed, and the gonadosomatic index (GI) was calculated for each fish (gonad weight proportional to the fish weight; see “Methods” for more details). Afterward, the testicular explants were treated, and histological slides of testis were made to account fish’s relative number of spermatozoa (see “Methods” for more details).

**Figure 5:**
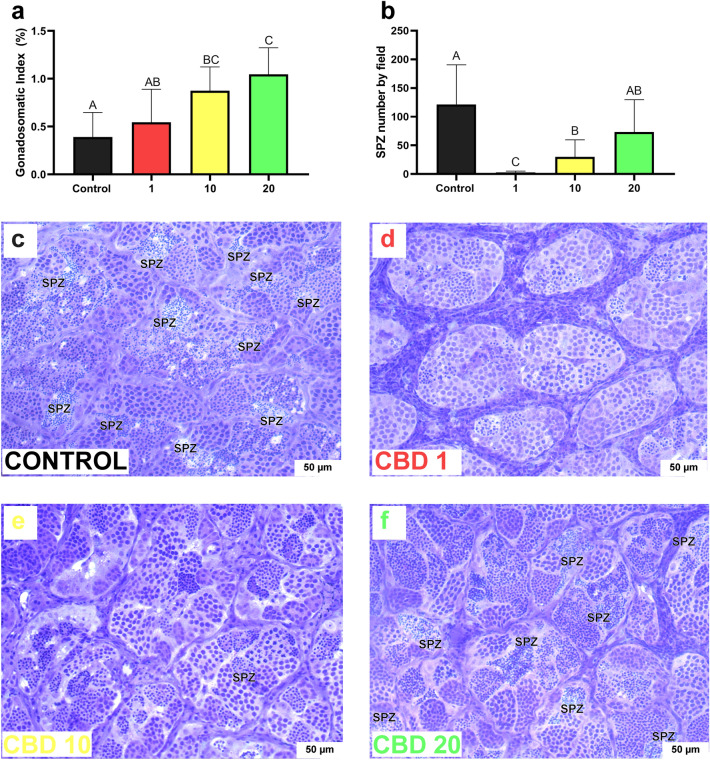
Ex vivo effects of 0 (control), 1 (CBD 1), 10 (CBD 10) and 20 mg/kg (CBD 20) of cannabidiol (CBD) on (**a**) gonadosomatic index (GI) of Nile tilapias (n = 10) and (**b**) the mean spermatozoa (SPZ) number by field (total of 20 fields by fish) in testicular explant from Nile tilapias (n = 5). Fish were exposed for 35 days to the CBD. Different uppercase letters indicate significant differences between treatments (Tukey HSD test, p < 0.05). The number of SPZs was determined by morpho-histological analysis using ImageJ software. Values are mean ± SEM. (**c**,**d**,**e**,**f**) Histological sections of Nile tilapia testis following 35-days of exposure to CBD. Spermatozoa areas are indicated by SPZ in each figure (scale: 50 μm).

Fish from CBD 10 and CBD 20 treatments presented a higher GI than those from control treatment. Moreover, fish from CBD20 treatment also presented a higher GI than fish from CBD 1 treatment (one-way ANOVA, F_3, 34_ = 11.121, p < 0.001; Tukey HSD test: CBD 10 vs. control: p = 0.003; CBD 20 vs. control: p < 0.001; CBD 20 vs. CBD 1: p = 0.003; Fig. 5a; Table 1). Although CBD have increased fish testis size, the drug significantly decreased the number of spermatozoa (Fig. 5b–f). CBD 1 and 10 fish presented a lower number of spermatozoa by field than control fish. Moreover, fish from CBD 1 treatment presented significantly less spermatozoa than any other CBD group (one-way ANOVA. F_3, 13_ = 18.588, p < 0.001; Tukey HSD test: Control vs. CBD 1: p < 0.001; control vs. CBD 10: p = 0.042; CBD 1 vs. CBD 10: p = 0.027; CBD 1 vs. CBD 20: p < 0.001; Fig. 5b; Table 1).

## Discussion

This study investigated CBD effects on behavioral and morpho-physiological variables related to Nile tilapia welfare. We found that the CBD 10 mg/kg dose efficiently reduced fish aggressiveness over 28 days. Regarding stress, CBD was not efficient in mitigating the stress responses of fish induced by a social stimulus. However, the CBD 20 mg/kg dose efficiently attenuated the VR increase induced by confinement stress (non-social stressor). Moreover, in the fifth week of the experiment, fish from CBD 10 and CBD 20 treatments presented lower baseline cortisol levels. No CBD dose affected feed ingestion or any growth variable of fish. In addition, the 10 and 20 mg/kg doses increased fish GI. Although fish testis size increased, doses of 1 and 10 mg/kg significantly reduced fish spermatozoa.

The CBD efficacy in decreasing animals’ aggressiveness was demonstrated only in mammals, specifically rats and dogs^34,35^, but not in other taxa so far. This study was the first to present evidence that oral CBD administration efficiently reduces fish aggressiveness. Aquaculture environments can increase Nile tilapia aggressive behavior^11^, which leads to higher social stress from social rank, more energy expenditure on fights, and an increase in the probability of body injuries, pain, infections and deaths^11^. Altogether, these factors severely compromise Nile tilapia’s welfare. Therefore, decreasing Nile tilapia’s aggressive behavior in artificial environments is essential to improving the welfare of this widely farmed fish species.

The intermediate CBD dose of 10 mg/kg was efficient in reducing fish aggressive behavior, while lower (1 mg/kg) and higher (20 mg/kg) doses were ineffective. In rats, intraperitoneal doses between 5 and 60 mg/kg of CBD decreased aggressiveness, being the doses of 15 and 30 mg/kg the most efficient^34^. The mechanism through which CBD decreases rats’ aggressive behavior is associated with activating 5-HT1A serotoninergic receptors and CB1 endocannabinoid receptors^34^. Although we did not investigate the mechanism involved in CBD anti-aggressive effects in fish, it was probably similar to the mechanism described in mammals since both receptors, 5-HT1A^57,58^ and CB1, are highly conservated between the taxa^50,51^ and play similar roles in aggressiveness decrease^59^. The activation of the endocannabinoid system by CBD and other cannabinoids often triggers biphasic effects in behavioral responses, such as feeding, locomotor activity and anxiety-like behavior^54^. The following scenarios characterize biphasic effects: low doses of a drug trigger an effect, while higher doses do not (or even trigger the opposite effect), and vice-versa^60^. The same classic CBD biphasic effect was probably found in our aggressive behavior results since the intermediate dose of 10 mg/kg decreased fish aggressiveness while the higher dose of 20 mg/kg did not.

The aggressive behavior in our study was accessed through the mirror test, a protocol widely used to assess individual aggressive behavior in fish^61–63^. The advantage of this protocol is allowing the access of the individual aggressive propensity without the influence of the opponent’s behavior, since in a dyadic fight, for example, the aggressive behavior depends on the individual as well as the aggressive motivation of the interactant^64,65^. However, there are some artificialities in the mirror test, as the image does not respond to the fighting individual, therefore generating criticism of this method. Despite this criticism addressed to the mirror test, it was recently shown that several cichlid species under natural environments react to their mirror image with meaningful biological behaviors, according to the aggressive behavioral pattern of the species, regardless of any artificiality of the method^66^. Moreover, the aggressive behavior in the mirror test was found in several studies to positively correlate with the aggressiveness level of fish in live agonistic trials^67,68^. Balzarini et al.^67^ compared mirror-elicited aggression with a live conspecific in three cichlid species and showed that similarities in behavior depend on the species. The authors suggested it is necessary to validate the methodology for each species before using mirror^67^. In this context, several studies with Nile tilapia used the mirror test and showed plausible results for comparing intraindividual aggression^7,63,69–71^. Therefore, although limitations on the method exist, we consider that our results are plausible and present evidence that CBD can decrease Nile tilapia’s aggressive behavior. This is the first time that the evidence has shown that CBD could decrease aggressiveness in non-mammal animals. Due to the high novelty character of the study, we encourage further studies to test whether the aggressive behavior decrease observed in our study is maintained in aquaculture conditions.

Regarding stress, CBD did not attenuate VR response to a social stimulus. However, concerning non-social stress, CBD effectively attenuated fish’s VR response. There may be multiple reasons for this to happen. First, the type of stressor (that is, if it is social or non-social) may influence the CBD stress-attenuation effect. Likewise, in humans, a 10 mg/kg CBD dose effectively decreased the heart rate and cortisol levels in response to a non-social stimulus (drug induction^72^), while the same dose was ineffective in attenuating the cortisol increase in response to a social stressor (“Trier Social Stress Test”) in patients with a high risk of psychosis^73^. Second, it is important to highlight that the time of exposure to CBD can also be a factor that interferes with its effects^74^. The stress in response to social stimulus was measured during the four initial experimental weeks, while the non-social stress was measured in the fifth week. Thus, we cannot discard that the time of exposure to CBD may also have played a key role in our results. Nevertheless, further studies are necessary to unravel the proper dosages and mechanisms behind CBD efficiency to mitigate different types of stress, primarily if the drug can mitigate social stress.

Besides the VR, we also evaluated the cortisol levels, the main stress hormone in fish^75^, in the fifth week of CBD exposure, at baseline levels, and after facing confinement (stress-induced levels in response to non-social stress). Even not decreasing the stress-induced cortisol levels of fish, CBD reduced baseline levels of the hormone, which is an essential factor in the welfare improvement of captive Nile tilapia and other species. Lower baseline levels of the hormone can be beneficial to organism functioning since higher cortisol levels for long periods lead to impairment of the reproduction system^76^, depress immunity^77^, and impair growth and organ development^78,79^, among other negative effects on the organism^79^. On the other hand, Mortuza et al. (2021)^80^ found that CBD did not significantly decrease other secondary stress biomarkers of Nile tilapia, such as glucose, haematocrit, and plasma protein in both stressed and non-stressed fish. The differences between our studies regarding the non-stressed cortisol levels are probably due to differences in the exposition time and doses. Mortuza et al.^80^ exposed Nile tilapias to small doses of CBD, in which the highest dose tested was similar to our smallest dose, 1 mg/kg (See “Methods”—Table 3), and for a short period of 3 days.

**Table 3:** General information about the cannabidiol (CBD) amount applied in each treatment diet (2 kg of ration per treatment).

Treatments (mg of CBD/kg of fish)	Initial fish weight (kg)	CBD amount applied to diet (mg)	CBD percentage on the ration (%)
Control	0.03 ± 0.003	0	0
CBD1 (1 mg/kg)	0.02967 ± 0.003	16.5	0.0033
CBD10 (10 mg/kg)	0.02867 ± 0.002	166.5	0.0333
CBD 20 (20 mg/kg)	0.02833 ± 0.003	333	0.0666

The CBD doses of each treatment (0, 1, 10 e 20 mg/kg) were calculated based on initial fish weight.

In “Initial fish weight”, values are mean ± SD.

*CBD* cannabidiol.

CBD can also affect food intake and body weight gain^33,45^. However, juvenile males of Nile tilapia supplemented with different CBD doses did not alter food intake or any of the measured growth response variables. It is important to highlight that diet supplemented with CBD was not unpalatable for fish, since all treatments ate statistically the same amount of food, and the method of CBD administration in the ration is valid for the taxon.

Although there was no CBD effect on growth variables, the 10 and 20 mg/kg doses increased fish’s gonadosomatic index (GI). The GI is a macroscopic measure used to estimate the gonadal maturation in several fish species^81,82^. The gonadal development can be impaired by high cortisol levels, decreasing the GI^83^. Thus, high GI values indicate good rearing conditions^84^ and signalize fish welfare state. Nile tilapias treated with high doses of CBD (10 and 20 mg/kg) had lower baseline cortisol levels in the fifth experimental week. Although the cortisol level was not accessed in the first four experimental weeks, perhaps it was already low and thus enabled fish testis increased development. Additional research can elucidate how long it takes for CBD to decrease the baseline cortisol to confirm or reject this hypothesis, and it may also investigate the CBD effect on other important hormones for the gonadal development, such as sexual hormones (e.g., testosterone, progesterone, and estradiol)^85^.

Although fish testis’ size increased with CBD treatment, there was no increase in spermatozoa number. Fish treated with 1 and 10 mg/kg of CBD presented fewer spermatozoa than control fish. In particular, the 1 mg/kg dose drastically decreased the number of spermatozoa cells (Fig. 5a,b). Probably, we observed another CBD biphasic effect in the number of spermatozoa. The biphasic nature of CBD was already observed concerning some male reproductive aspects, such as testosterone bioavailability and sexual behavior^40^. Other studies did not find CBD effect on the number of spermatozoa^86^, but reported deregulation on spermatogenesis, impairment of sperm quality^40,86^, motility^37,47,86^, and also reduction in sperm fertility^37,87^ in several animal species (sea urchins, CBD concentration of 0.1–10 μM^87^; rats, 15 and 30 mg/kg^40^; and zebrafish, CBD concentration of 0.5 μM^47^). However, there is no general agreement regarding the reversibility or irreversibility of the reproductive toxicity effects of CBD on the male reproductive system^40^. It seems that the period of exposure to the drug can lead to reversible or irreversible effects of CBD on the reproductive system^40^. Exposures during gonadal development can lead to irreversible and long-term effects^40,88^, while exposures after this period lead to reversible effects^37,40^. Therefore, to promote CBD as a tool to improve animal welfare, these toxic effects on males’ reproductive system must be considered. In addition, there must be a better comprehension of whether these effects are reversible or irreversible and at what age the use of CBD is safe for the male reproductive system.

Finally, CBD seems to be a promising tool for increasing Nile tilapia welfare in captive environments since, depending on the dose (10 or 20 mg/kg), the drug decreases animals’ aggressiveness (10 mg/kg), non-social stress (20 mg/kg), and baseline cortisol levels (10 and 20 mg/kg), besides increases testis size (10 and 20 mg/kg). CBD can easily be offered in fish diets, and the 20 mg/kg can be more suitable to improving the welfare of non-aggressive social fish species, while the 10 mg/kg dose can be used to improve the welfare of aggressive fish species, such as the Nile tilapia. However, it is necessary to be cautious when using the 10 mg/kg dose for Nile tilapias reared for reproduction purposes since it significantly reduces fish spermatozoa. We understand that this is the first time that evidence has been shown of CBD’s potential as a tool to improve the welfare of captive and farmed animals. We highlight that CBD can improve Nile tilapia welfare and does not reduce fish growth. These results provide a win-win situation for both animals, which will have life quality, and fish farmers, who can keep their production while adding value to their product. Currently, CBD is not a cheap resource, but low concentrations, as used in this study, have already presented promising results. Besides, *Cannabis* is increasingly being socially accepted, and there is a trend for legalization in many countries. Thus, the prices may decrease in the near future^80^. In this study, we used a laboratory approach, isolating fish to obtain the CBD’s effect on animals’ behavior, physiology, and morpho-histological aspects related to welfare. Thus, before implementing CBD in fish farms, further studies are necessary to test whether the welfare improvement observed in our study is maintained in aquaculture conditions, if CBD affects the fish meat quality and if there is an accumulation of the drug in the meat and water.

## Methods

### Animals and experimental conditions

In aquaculture, typically only male Nile tilapias were reared in tanks, since males present a higher growth rate than females^89^. Thus, juveniles of Nile tilapia (Supreme strain and sexually reversed—all males, around 4 months age) were obtained from fish farming at Botucatu–SP, Brazil, and used to constitute a stock population (500L tank, 100 fish). Fish were maintained in the stock tank for one month and were fed with a commercial diet (Presence Nutripiscis—extruded ration, pellets of 3–4 mm) corresponding to 3% of their body weight fractionated at three meals in a day (9:00 h, 13:00 h, and 17:00 h). The stock tank was equipped with constant aeration and a biological filter, which helped to maintain water quality parameters (pH: 6.8–7.2, ammonia ≤ 0.05 ppm, and nitrite ≤ 0.5 ppm; water temperature 25–27 ℃). After this period, 60 fish were selected for the experiment beginning and were individualized in 23L aquaria (40 x 23 x 25 cm) without visual contact (aquaria are separated by opaque partitions). Nile tilapias were randomly assigned to one of the four treatments (control, CBD1, CBD10 and CBD20; n = 15/group). Aquaria’s water ranged between 25 and 27 ℃ and was supplied with constant aeration, and a 12-h light cycle (6:00 to 18:00 h) was set. Water was partially changed (40%) every 2 days to maintain quality parameters (pH: 6.8–7.2, ammonia ≤ 0.05 ppm, and nitrite ≤ 0.5 ppm). Fish were fed with a treated commercial diet (Presence Nutripiscis—extruded ration, pellets of 3–4 mm) corresponding to 3% of their body weight fractionated at three meals in a day (9:00 h, 13:00 h, and 17:00 h). At the beginning of the experiment, the average fish weight was 29.5 ± 2.8 g (mean ± SD). All tests were conducted at 8:00–12:00 h with fasted animals (fasted for 15 h).

### Cannabidiol and treated ration preparation

The CBD used in this study was isolated in salt (99% concentration) and was obtained in collaboration with Prof. Elisaldo Carlini from the Universidade Federal de São Paulo (UNIFESP). CBD is a highly liposoluble drug^90^; therefore, to add it to the fish diet, the drug was diluted in soybean oil, and a top coating with this mix was applied to commercial pellets.

The CBD dose of each treatment (0 mg/kg, 1 mg/kg, 10 mg/kg, and 20 mg/kg) was calculated based on fish’s initial weight (approximately 30 g; Table 3) without adjustments through all experiment. The total CBD amount applied to each treated diet (Table 3) was calculated based on the following formula:

$$CBD \,amount\, applied\, to\, the\, treated\, diets\,=\, CBD\, dose\, \left(mg\right)\times\, 15\, fish\times\, 35\, days$$

In which 15 is the N of fish per treatment and 35 days the experimental period.

The treated rations preparation was conducted in a room without light due to CBD photosensitive property^91^, and 2 kg of pellets were used for each treatment. The total CBD amount of each treated diet (Table 3) was dissolved in a quantity of soybean oil corresponding to 2% of 2 kg of ration. To dissolve CBD in soybean oil, a heat magnetic stirrer (Biomixer AM-10), at 60 ℃, rotating at 3000 rpm was utilized. Afterward, the pellets were placed homogeneously on a straight surface, and the oil with CBD was applied to it with a hand sprayer. To ensure that CBD was applied in all superficies, the pellets were turned four times during oil application, and after that, they were put in a plastic bag and shaken for 3 min. Lastly, the pellets were placed on a surface and left out at room temperature for 1 day to dry. The same procedure was done to prepare the control group diet, however, without CBD addition.

### Mirror test—Aggressive behavior and response to a social stimulus

The mirror test was used to assess fish’s aggressive behavior and stress response to a social stimulus in the first 4 weeks (Fig. 1). Mirror-elicited fighting (the mirror test) is a reliable method to evaluate individual fish aggressive behavior in several species^61,62^, including the Nile tilapia^63,69^. Cichlid fishes (for example, the Nile tilapia) cannot recognize their image in the mirror, perceiving it as another conspecific entering their territory. Thus, they exhibit aggressive interactions against the mirror^92^. This type of protocol allows us to evaluate the individual aggressive propensity as there are no wins or losses^93,94^; therefore, no effect of social rank on the stress response^95,96^. Additionally, mirror-elicited aggression is a predictor of individual stress^70^ and is associated with gene expression in the hypothalamus of critical genes involved in the stress response of Nile tilapia^71^. Furthermore, despite criticism addressed to this method, it was recently demonstrated that several cichlid species in their natural environments aggressively react to their mirror image, expressing resembling behavioral patterns presented during fights with co-specifics^66^.

In total, five mirror tests were performed. The first one was performed on day 1 before fish started to receive the treated diet to assess the basal fish’s aggressiveness level and stress response (Fig. 1). Afterward, fish started to receive the treated diets, and mirror tests were performed once per week (Week 1, Week 2, Week 3, and Week 4) (Fig. 1). In the mirror test, each fish was recorded for 10 min (n = 15). On one side of the aquarium, a mirror of the same size as the lateral wall was placed parallel to this, covered by an opaque divisor. The recording started when the opaque divisor was removed. The latency for the fish to perform the first aggressive behavior against the mirror and the frequency of attacks against the mirror (bites, touches and lateral fights^63,97^) were visually accounted for according to the ethograms described for the species^63,97^ by an experimenter blind to fish’s treatment. Fish that did not attack the mirror in any of the sample time points were excluded from the analysis.

Moreover, the VR pre- and post-social stimulus (mirror test) were measured (n = 15) to calculate the ΔVR. The VR is a physiological response of fish and a reliable measure of stress level since it increases in response to stressors and is related to the metabolic rate^98^. To measure the VR, the time that fish took to perform 20 successive opercular or buccal movements was accounted^98^. Next, it was calculated how many opercular beats per minute each fish would execute through an estimate. Both VRs (pre- and post-social stimulus) were measured 3 times per fish in three consecutive minutes. The mean for each fish was calculated^98^ and used to obtain the ΔVR in response to a social stimulus, our dependent variable. The individual variation of VR in response to the mirror test, or ΔVR, was calculated through the following formula: ΔVR = VR post-social stimulus– VR pre-social stimulus.

### Stress responses to a non-social stimulus—confinement stress

In the experiment’s fifth week, some fish’s stress responses (specifically, cortisol level and VR) to non-social stress were measured. On the first day of the week (Experimental Day 29, Fig. 1), blood sampling for cortisol assay (baseline levels) was performed through cardiac puncture using heparinized syringes. On the last day of the week (Experimental Day 35, Fig. 1), confinement stress (non-social stimulus) was applied to fish. To this end, an opaque partition was used to restrict fish to only 10% of the aquarium volume for 30 min. The VRs pre- and post-confinement were measured (n = 14, since some fish died before the first blood sampling) and used to calculate the ΔVR in response to confinement (ΔVR = VR post-confinement– VR pre-confinement). Moreover, a second blood sampling for cortisol assay (stress-induced levels) was performed through cardiac puncture using heparinized syringes 30 min after confinement^99^.

### Cortisol assay

Fish were taken from their aquaria with an aquarium dip net (n = 8), anaesthetized by immersion in an aquarium with clove oil (280 mg/l), and posteriorly a blood sample was collected (0.4 ml) through cardiac punction with hypodermic needles and heparinized syringes. After this procedure, fish were returned to their aquaria. The handling time between removing and returning fish to the aquarium lasted less than 5 min for all individuals to avoid any bias of handling time in the cortisol levels. The blood was centrifuged at 3000 rpm for 10 min to separate approximately 0.1 ml of plasma, which was frozen at – 20 ℃ for further cortisol assays. The cortisol was measured by Enzyme Linked Immunosorbent Assay (ELISA), using commercial cortisol kits (DRG, Marburg, Germany).

### Feed ingestion and growth variables

Daily, 39 pellets were offered to each fish (approximately 0.9 g—equivalent to 3% of animals’ initial weight), divided into three meals (9:00 h, 13:00 h, and 17:00 h; 13 pellets per meal). After a meal, the pellets left in each aquarium were accounted for and removed right before the next meal. The daily feed ingestion was calculated by subtracting the number of pellets left of 39 (total offered in a day). Afterward, the consumed mass of feed in a day was inferred from this value. Lastly, the consumed mass of feed each week was calculated for further analysis.

At the end of the fourth week, fish were weighed, and the following growth variables were measured (n = 15): average weight gain (AWG), feed conversion (FC), specific growth rate (SGR), and condition factor (K). The AWG, FC, SGR and K were calculated through the following formulas, respectively:

$$AWG\,=\,Final\, weight\, (g)\,-\,Initial\, weight\, (g)$$

$$FC\,=\, \frac{Feed\, consumption\, (g)}{AWG}$$

$$SGR\,=\,\frac{\mathrm{ln}Final\, weight\, \left(g\right)\,-\,\mathrm{ln}\,Initial\, weight\, (g)}{Experimental\, period\, (days)}\, \times\, 100$$

$$K=\frac{Final\, Weight\, (g)}{{Final\, standard\, length\, (cm)}^{3}} \times 100$$

### Morpho-histological analysis of fish’s reproductive system

At the end of the fifth experimental week, testis were collected for morphological and histological analyses. Right after being collected, the gonads were weighed, and the gonadosomatic index (GI) (n = 10) was calculated following the formula:

$$GI=\frac{Gonad\, weight\, \left(g\right)}{Fish\, weight\, \left(g\right)}\times\, 100$$

Afterwards, the testicular explants were fixed in 4% Karnovsky at 4 ℃ for at least 24 h, dehydrated and embedded in a Technovit (7100) historesin (Heraeus Kulzer, Germany). Subsequently, the samples were sectioned at 3 µm of thickness and stained with 0.1% toluidine blue. The histological sections obtained were used to quantify the relative number of spermatozoa of fish (n = 5). Twenty non-overlapping fields were randomly chosen and photographed using a Leica DMI6000 microscope (100 × objective lens total magnification). The ImageJ software^100^ was used to account for the number of spermatozoa in each field^101,102^. The mean spermatozoa number by field was calculated for each fish for further analysis.

### Statistical analysis

All the statistical analyses were performed in the R environment software (v3.6.0.)^103^. Shapiro-Wilk and Levene tests were used to test the normality and homoscedasticity of residuals, respectively, for the stress response variables measured in the fifth week (ΔVR in response to confinement, cortisol baseline, and stress-induced levels), growth variables (AWG, FC, SGR and K) and morpho-histological variables of reproduction (GI and spermatozoa number by field). An ANOVA one-way was performed when the data met the parametric assumptions, and a Kruskal–Wallis test was done when data did not meet any parametric assumption. All the response variables mentioned above were predicted by “treatment,” a four-level (levels: control, CBD 1, CBD 10, CBD 20) categorical independent variable. Post-hoc comparisons were performed using the Tukey HSD test. Moreover, it is important to highlight that the cortisol baseline and stress-induced values were analyzed separately by two distinct ANOVAs one-way, one for each variable, instead of analyzing both variables in a unique LMM. This strategy was adopted since the baseline cortisol level was collected one week before the confinement stress, and the stress-induced cortisol level was collected 30 min after the confinement. Baseline cortisol levels may vary over days^75,104,105^ and the hours within a day in fish^75,99^. Thus, the baseline level one week ago could not be the same one week later on the confinement day, and comparing these measures could not make much sense. More authors adopted the same approach to analyze the baseline and stress induces cortisol levels separately^75,104^.

Regarding the following response variables: aggressive behavior (number of attacks and latency for the first aggressive behavior), stress responses to a social stimulus (ΔVR in response to a social stimulus), and feed ingestion, LMM were performed. The independent categorical variable “treatment” (levels: control, CBD 1, CBD 10, CBD 20) and “sampling time points” (e.g., levels: basal, Week 1, Week 2, Week 3 and Week 4) were set as fixed factors in the models, and “fish ID” was included as a nested random factor. The normality of residuals assumption was checked through visual analysis of Normal quantile-quantile plots (QQ plots) of residuals (using the “qqnorm” function in R) and also the Shapiro-Wilk test. The assumption of linearity and homoscedasticity of residuals were graphically evaluated through the “plot” function in R. The assumptions were valid for all mixed models built on this study. Post-hoc comparisons were performed using the Tukey HSD test. The significance level for all statistical tests performed in this work was set at α = 0.05. All the data supporting the results of this paper is available as supplementary material (see Supplementary material for more details).

### Ethical note

The current research was conducted in accordance with the Ethical Principles on Animal Experimentation adopted by the National Council for the Control of Animal Experimentation (CONCEA/Brazil). All procedures used in this study were approved by the CEUA (Committee on Ethics in the Use of Animals) of São Paulo State University (UNESP), protocol # 4166190321, and reporting follows the recommendations in the ARRIVE guidelines.

## Supplementary Information

Supplementary Figure 1.

Supplementary Figure 2.

Supplementary Figure 3.

Supplementary Figure 4.

Supplementary Figure 5.

Supplementary Figure 6.

Supplementary Table 1.

Supplementary Table 2.

## Data Availability

The data that support the findings of this study are available from the corresponding author, B.C-d-S., upon request.
